# A key to the bamboo-feeding genus *Bambusana* Anufriev (Hemiptera, Cicadellidae, Deltocephalinae, Athysanini), with description of one new species from China

**DOI:** 10.3897/zookeys.861.34811

**Published:** 2019-07-08

**Authors:** Qiang Luo, Ya-Lin Yao, Lin Yang, Xiang-Sheng Chen

**Affiliations:** 1 Institute of Entomology, Guizhou University, Guiyang, Guizhou, 550025,China Guizhou University Guiyang China; 2 The Provincial Special Key Laboratory for Development and Utilization of Insect Resources, Guizhou University, Guiyang, Guizhou, 550025, China Guizhou University Guiyang China; 3 The Provincial Key Laboratory for Agricultural Pest Management of Mountainous Regions, Guizhou University, Guiyang, Guizhou, 550025, China Guizhou University Guiyang China

**Keywords:** Homoptera, morphology, new species, taxonomy

## Abstract

A new species of the bamboo-feeding leafhopper genus *Bambusana* Anufriev, 1969, *B.longispina* Luo & Chen, **sp. nov.** is described and illustrated from China (Yunnan Province). A checklist and key to known species of this genus are provided. Figures are also provided for *B.bambusae*, *B.biflaka*, *B.fopingensis* and *B.multidentata*.

## Introduction

The leafhopper genus *Bambusana* (Deltocephalinae, Athysanini) was established by Anufriev (1969) with two species: *B.bambusae* (Matsumura, 1914) (type species) and *B.jenjouristi* Anufriev, 1969 from Japan. Later, Anufriev and Emeljanov (1988) reported *B.bambusae* from the Soviet Far East. Dai and Zhang (2006) first recorded this genus from China and described two new species: *B.fopingensis* and *B.multidentata*, and reported *B.bambusae* from China. Recently, Li (in Li et al. 2011) described two new species from China: *B.biflaka* and *B.nigrimaculata*, and recognized *B.fopingensis* as a junior synonym of *B.multidentata* in their study but did not provide any justification for the synonymy. We here still recognize *B.fopingensis* as a valid species based on the pygofer with a strong ventro-caudal process which is significantly different from *B.multidentata*.

In this paper, a new species, *B.longispina* sp. nov. is described and illustrated from Yunnan Province, China bringing the total in the genus to seven (six from China); see key.

## Material and methods

The terminology of morphological and genital characters follows Li et al. (2011) and Zahniser and Dietrich (2013). Male specimens were used for the descriptions and illustrations. External morphology was observed under a stereoscopic microscope and characters were measured with an ocular micrometer. Color pictures for adult habitus were obtained by using the KEYENCE VHX-1000 system. The genital segments of the examined specimens were macerated in 10% NaOH and drawn from preparations in glycerin jelly using a Leica MZ 12.5 stereomicroscope. Illustrations were scanned with a Canon CanoScan LiDE 200 and imported into Adobe Photoshop CS8 for labeling and plate composition.

The type specimens of the new species are deposited in the Institute of Entomology, Guizhou University, Guiyang, China (IEGU).

## Taxonomy

### Genus Bambusana

Taxon classificationAnimaliaHemipteraCicadellidae

Anufriev, 1969

Figs 1–6 , 7–15 , 16–20 , 21–35 , 36–51

Bambusana Anufriev, 1969: 403; Dai and Zhang 2006: 63; Li et al. 2011: 40.

#### Type species.

*Thamnotettixbambusae* Matsumura, 1914, by original designation.

#### Diagnosis.

This genus can be differentiated from other genera of Athysanini by the follow characters: relatively elongate leafhoppers with crown slightly longer medially than next to eyes; male pygofer side elongate, with one or two well sclerotized processes on ventral margin; subgenital plate usually elongate, triangular; aedeagus with basal apodeme usually present, shaft with or without small distal processes; gonopore apical or apical on ventral surface.

#### Description.

**Figures 1–6.**
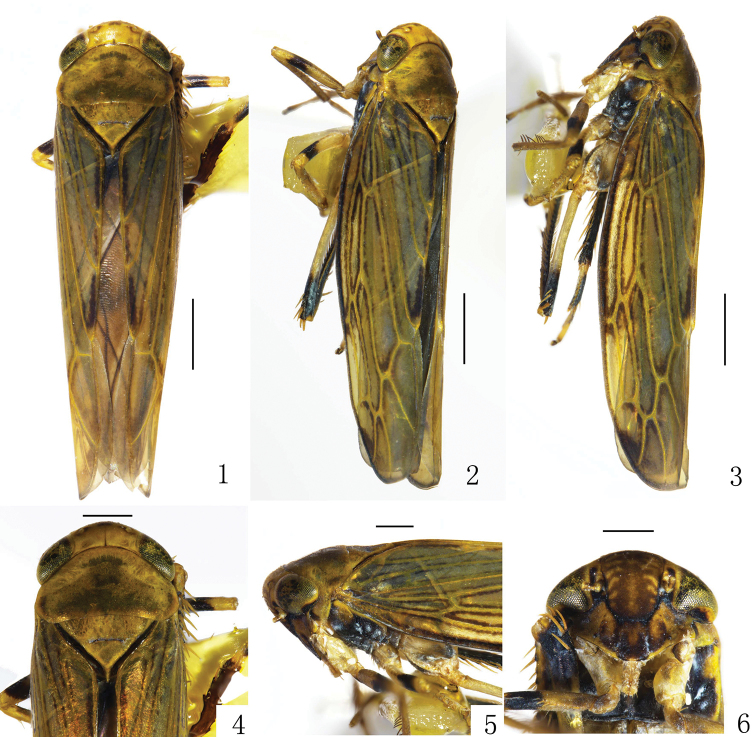
*Bambusanalongispina* sp. nov. **1** Male habitus, dorsal view **2** male habitus, dorsal and lateral view **3** male habitus, lateral view **4** head and thorax, dorsal view **5** head and thorax, lateral view **6** face. Scale bars: 1.0 mm (**1–3**); 0.5 mm (**4–6**).

Body elongate. Head including eyes subequal to or slightly wider than pronotum (Figs 1, 21, 24, 27, 30, 33). Crown with anterior margin roundly produced anteriorly, distinctly shorter medially than width between eyes (Figs 1, 21, 24, 27, 30, 33). Transition of crown to face rounded (Figs 5, 22, 25, 28, 31, 34); ocellus situated on or near frontal lateral margin of crown, less than 1/3 distant from eye to crown apex (Figs 1, 21, 24, 27, 30, 33). Clypellus widening apically, relatively flat (Figs 6, 23, 26, 29, 32, 35). Pronotum with anterior margin strongly and roundly produced, posterior margin slightly concave. Scutellum subequal to or slightly shorter than pronotum (Figs 1, 4, 22, 25, 28, 31, 34). Forewing elongate and rounded apically, considerably longer than abdomen, with four apical cells; appendix well developed (Figs 1–3, 21, 22, 24, 25, 27, 28, 30, 31, 33, 34).

Male genitalia with pygofer elongate in profile, with one or two sclerotized processes on ventral margin or ventral margin dentate; several macrosetae posteriorly (Figs 7, 8, 36, 37, 40, 41, 44, 45, 48, 49). Valve narrowly triangular, subequal to or shorter than length of subgenital plate (Fig. 15). Subgenital plate elongate, triangular, a uniseriate row of macrosetae along ventrolateral margin (Fig. 15). Connective Y-shaped, shaft subequal to or distinctly longer than arms (Fig. 13). Styles elongate, apical process short to long, tapered to acute apex; lateral lobe weakly or well developed, with a few fine setae (Figs 12, 14). Aedeagus with basal apodeme usually present, shaft elongate, cylindrical, with or without small process near apex, gonopore small, apical or apical on ventral surface; with short preatrium sometimes present (Figs 9–11, 38, 39, 42, 43, 46, 47, 50, 51).

**Figures 7–15.**
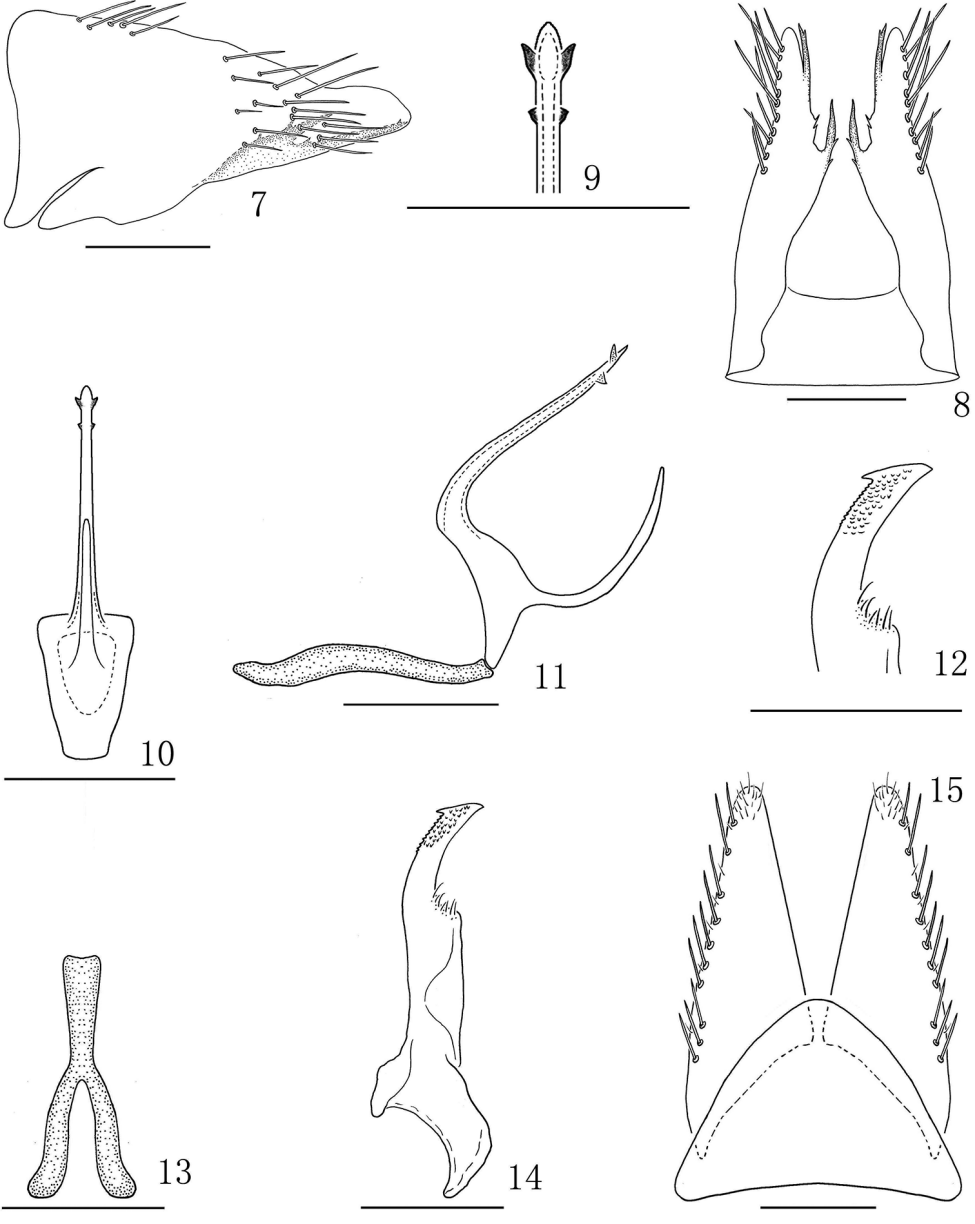
*Bambusanalongispina* sp. nov. **7** Male pygofer, lateral view **8** male pygofer, ventral view **9** apex of shaft, ventral view **10** aedeagus, caudal view **11** aedeagus and connective, lateral view **12** apex of style, dorsal view **13** connective, dorsal view **14** style, dorsal view **15** valve and subgenital plate, ventral view. Scale bars: 0.2 mm (**7–15**).

**Figures 16–20.**
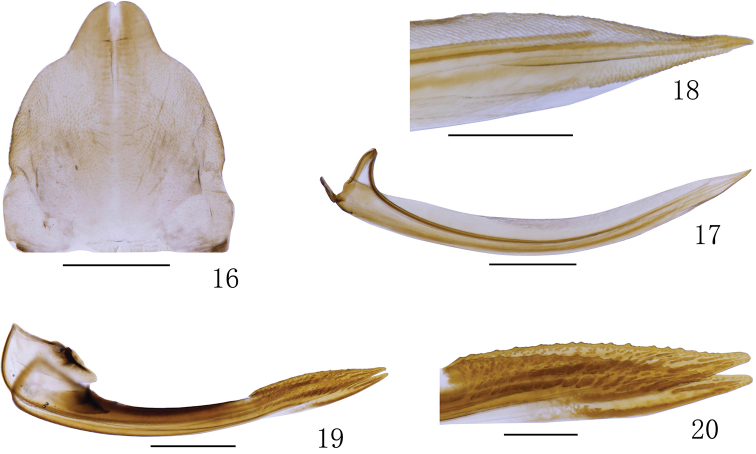
*Bambusanalongispina* sp. nov. **16** Female sternite VII, ventral view **17** first valvula, lateral view **18** apex of first valvula, lateral view **19** second valvula, lateral view **20** apex of second valvula, lateral view. Scale bars: 0.5 mm (**16–17, 19**); 0.2 mm (**18, 20**).

**Figures 21–35.**
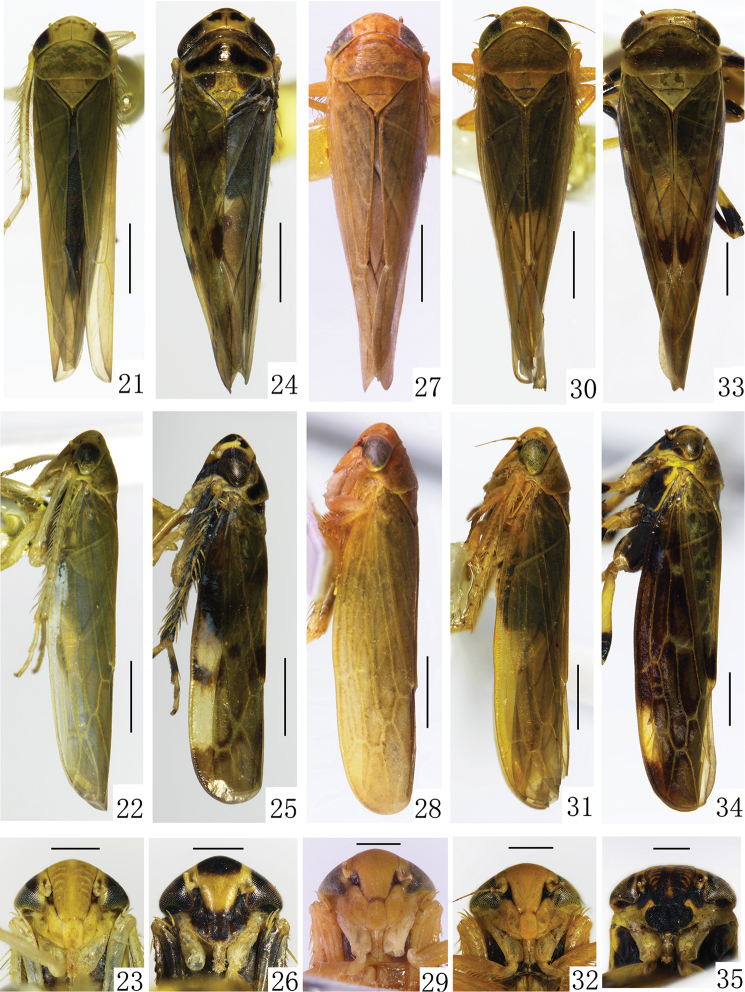
**21–23***B.bambusae***21** male habitus, dorsal view **22** male habitus, lateral view **23** face **24–26***B.biflaka***24** male habitus, dorsal view **25** male habitus, lateral view **26** face **27–29***B.fopingensis***27** male habitus, dorsal view **28** male habitus, lateral view **29** face **30–32***B.multidentata***30** male habitus, dorsal view **31** male habitus, lateral view **32** face **33–35***B.nigrimaculata***33** male habitus, dorsal view **34** male habitus, lateral view **35** face. Scale bars: 1.0 mm (**21, 22, 24, 25, 27, 28, 30, 31, 33, 34**); 0.5 mm (**23, 26, 29, 32, 35**).

**Figures 36–51.**
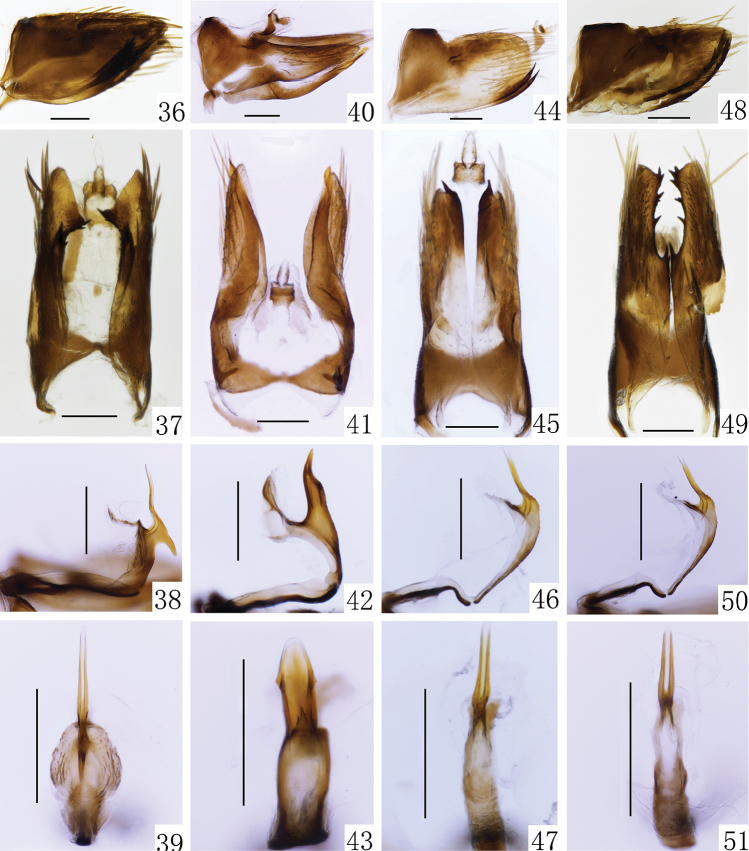
**36–39***B.bambusae***36** male pygofer, lateral view **37** male pygofer, ventral view **38** aedeagus and connective, lateral view **39** aedeagus, caudal view **40–43***B.biflaka***40** male pygofer, lateral view **41** male pygofer, ventral view **42** aedeagus and connective, lateral view **43** aedeagus, caudal view **44–47***B.fopingensis***44** male pygofer, lateral view **45** male pygofer, ventral view **46** aedeagus and connective, lateral view **47** aedeagus, caudal view **48–51***B.multidentata***48** male pygofer, lateral view **49** male pygofer, ventral view **50** aedeagus and connective, lateral view **51** aedeagus, caudal view. Scale bars: 0.2 mm.

#### Distribution.

China; Japan; Russia.

##### Checklist and distributions of species of *Bambusana* Anufriev, 1969

*B.bambusae* (Matsumura, 1914), Anufriev 1969: figs 1–6; Dai and Zhang 2006: figs 14–19; Li et al 2011: figs 5–29(1–6). China (Guizhou, Henan, Gansu); Japan (Hokkaido, Honshu, Shikoku); Russia

*B.biflaka* Li, 2011: figs 5–31(1–7), II-1. China (Sichuan, Hainan)

*B.fopingensis* Dai & Zhang, 2006: figs 1–8. China (Shaanxi, Guizhou)

*B.jenjouristi* Anufriev, 1969: figs 7–11. Japan (Honshu, Kobe)

*B.longispina* Luo & Chen, sp. nov. China (Yunnan)

*B.multidentata* Dai & Zhang, 2006: figs 9–13. China (Guizhou, Shaanxi)

*B.nigrimaculata* Li, 2011: figs 5–32(1–7), II-2. China (Yunnan)

##### Key to species of *Bambusana* (males only)

**Table:** 

1	Pygofer with two sclerotized processes on ventral margin	**2**
–	Pygofer with one sclerotized process on ventral margin or ventral margin dentate	**3**
2	Aedeagus with shaft with a tooth-like ventro-basal process, directed ventrally, without small processes subapically (Figs 38, 39)	*** B. bambusae ***
–	Aedeagus with a subbasal medial process from ventral margin, directed dorsally; shaft with a pair of subapical processes on each side (Figs 9–11)	***B.longispina* sp. nov.**
3	Pygofer with a long process arising from base of ventral margin, shaft with one pair of small processes near apex	**4**
–	Pygofer with one sclerotized process or ventral margin dentate, shaft without process near apex	**5**
4	Aedeagal shaft nearly straight in lateral view (Fig. 42)	*** B. biflaka ***
–	Aedeagal shaft sinuate in lateral view (Li et al. 2011: fig. 5-32-5)	*** B. nigrimaculata ***
5	Pygofer with a process arising from middle of ventral margin; aedeagal shaft with a tooth-like process subbasally (Anufriev 1969: figs 7, 10, 11)	*** B. jenjouristi ***
–	Pygofer with a strong ventro-caudal process or ventral margin dentate; aedeagal shaft without process subbasally	**6**
6	Pygofer with ventral margin dentate; style with lateral lobe well developed (Figs 48, 49, Dai and Zhang 2006: figs 9–11)	*** B. multidentata ***
–	Pygofer with a strong ventro-caudal process, style with lateral lobe weakly developed (Figs 44, 45, Dai and Zhang 2006: figs 4, 5)	*** B. fopingensis ***

### Bambusana longispina

Taxon classificationAnimaliaHemipteraCicadellidae

Luo & Chen sp. nov.

http://zoobank.org/141DEC67-E295-4678-B247-35D9AFC29B39

Figs 1–6 , 7–15 , 16–20

#### Description.

*Measurements.* Body length (including forewing): male 6.76 mm (1 specimen); female 6.80 (1 specimen); forewing length: male 5.60 mm (1 specimen); female 5.72 (1 specimen).

*Coloration.* Generally yellowish brown (Figs 1–3). Crown and pronotum yellowish brown to brown (Figs 4, 5). Face dark brown (Fig. 6). Legs with dark spots (Fig. 3). Forewing with yellow veins (Figs 1–3).

*Head and thorax.* Head including eyes as long as width of pronotum. Crown with fore margin arc-shaped, median length shorter than width between eyes (0.4:1) (Figs 1, 4). Coronal suture visible at basal one-third to one-half of crown (Figs 1, 4). Ocelli about 1/3 distant from eye to crown apex (Figs 1–4). Face with frontoclypeus longer than wide; anteclypeus slightly expanded apically; lorum broad (Fig. 6); antennae arising near lower corner of eye (Fig. 6). Scutellum slightly shorter than pronotum (0.8:1) (Fig. 4). Forewing elongate, with four apical cells; appendix wide (Figs 1–3).

*Male genitalia.* Pygofer elongate in profile, ventral margin with two elongate acute processes at distal one-third and subapically (Figs 7, 8), with a few fine teeth-like processes along ventroposterior margin (Fig. 8). Valve triangular, basal width slightly longer than median length (1.42:1) (Fig. 15). Subgenital plate elongate, triangular; with uniseriate row of ventral macrosetae along lateral margin; apical margin rounded with very short fine setae (Fig. 15). Connective Y-shape, shaft robust, similar length to arms (Fig. 13). Styles (Figs 10, 13) elongate, with apophysis relatively long and stout with small subapical tooth-like process from inner margin (Figs 12, 13). Aedeagus with basal apodeme absent; shaft elongate, cylindrical, tapering to acute apex, with two pairs of small triangle processes near apex (Figs 9–11); with subbasal elongate medial process from ventral margin, directed dorsally; with short preatrium.

*Female genitalia.* Sternite VII (Fig. 16) with anterior margin nearly straight and posterior margin strongly convex with blunt median tooth. First valvula (Figs 17, 18) curved, tapering apically with strigate sculpture extended to dorsal margin. Second valvula (Figs 19, 20) broad, gradually tapered to acute apex; dorsal margin with numerous small triangular teeth; with dorsal sclerotized and hyaline region.

#### Type material.

***Holotype***: ♂, **China**: Yunnan Province, Maguan County, 23. XI. 2016, Ya-Lin Yao. ***Paratype***: 1♀, same as holotype.

#### Host plants

. Bamboo.

#### Distribution.

Southwest China (Yunnan Province).

#### Remarks.

This new species is similar to *B.bambusae*, but can be distinguished from the latter by: aedeagus with a long medial process subbasally from ventral margin, directed dorsally (Figs 10, 11) (aedeagus with a tooth-like ventro-basal process, directed ventrally in *bambusae*); shaft long and tapered to apex, with two pairs of small triangle processes near apex (Figs 9–11) (shaft without process subapically in *bambusae*).

#### Etymology.

The name is derived from the Latin words “longus” and “spina”, referring to the aedeagus with a long spinous process near base (Fig. 11).

## Supplementary Material

XML Treatment for Bambusana

XML Treatment for Bambusana longispina
